# Physiotherapists’ awareness of risk of bone demineralisation and falls in people living with HIV: a qualitative study

**DOI:** 10.1186/s12913-021-06343-1

**Published:** 2021-04-13

**Authors:** Maria Y. Charumbira, Karina Berner, Quinette Louw

**Affiliations:** Division of Physiotherapy, Department of Health and Rehabilitation Sciences, Faculty of Medicine and Health Sciences, Stellenbosch University, P.O. Box 241, Cape town, 8000 South Africa

**Keywords:** Accidental falls, Bone mineral density, HIV, Physiotherapists, Sub-Saharan Africa

## Abstract

**Background:**

Recent research has indicated a seemingly increased propensity for both falls and accelerated bone loss in people living with HIV (PLWH). Physiotherapists play a crucial role in optimising function and quality of life of PLWH through prevention of falls and reducing the harm that results.

**Aim:**

This study aimed to explore physiotherapists’ awareness of falls risk and accelerated bone demineralisation in PLWH and their perceptions of current falls prevention strategies in the care of PLWH in selected regions of sub-Saharan Africa.

**Method:**

An exploratory descriptive qualitative research method was employed to explore physiotherapists’ perceptions and experiences regarding bone health and falls in PLWH. In-depth semi-structured telephonic interviews were used to collect data from 21 physiotherapists working in primary HIV care. Transcribed interview data were coded in Atlas.ti.8® and analysed using inductive thematic analysis.

**Results:**

The primary study revealed a lack of awareness by physiotherapists of falls risk and bone demineralisation in PLWH. As such, physiotherapists did not link falls or fractures to HIV or antiretroviral therapy (ART) when they did observe such events during their general patient assessments. However, in retrospect, some physiotherapists were able to recognise risk factors linked to falls in those with HIV. Current services for falls prevention, as perceived by the physiotherapists, were sub-optimal.

**Conclusion:**

Physiotherapists may need to be more aware of the potential risk of falls and bone demineralisation in PLWH and routinely assess for these phenomena in both older and younger PLWH.

**Supplementary Information:**

The online version contains supplementary material available at 10.1186/s12913-021-06343-1.

## Introduction

Falls among people living with HIV (PLWH) is an emerging concern. The first study was published recently, in 2012 [1]. Most research, done in high income countries, indicates that reduced bone mineral density (BMD), increased risk of falls and fractures are associated with antiretroviral therapy (ART) exposure, HIV infection itself and traditional risk factors [2–4]. As affordable ART became readily accessible in lower middle-income countries (LMICs), there was a need for more sub-Saharan African data and evidence to guide HIV care within a rehabilitation framework so that PLWH may not only live longer, but do so with improved quality of life (QoL).

The scope of physiotherapy practice in the rehabilitation of PLWH in the ART era is progressively being understood by physiotherapists in LMICs [5, 6]. Randomised controlled trials in Southern Africa have proved the efficacy of physiotherapy on pain management, cardio-pulmonary fitness, strength, and QoL in PLWH [7, 8]. However, the role of physiotherapy in health promotion and prevention in PLWH is not well understood [9]. Scant evidence exists for physiotherapy interventions that reduce falls [10] or promote bone health [11, 12] in PLWH and the role of physiotherapy for HIV at primary care is not clearly understood by the interdisciplinary team [6]. This may result in inappropriate, delayed or non-referral of PLWH who may have benefited from physiotherapy; as well as physiotherapists being side-lined from HIV care policy-making dialogues [13]. Physiotherapists need to be aware of their scope of practice in primary HIV care, particularly falls and fracture prevention, before they can promote it among other health professionals and the community at large [6].

A knowledge gap exists regarding physiotherapists’ awareness of falls and bone demineralisation in PLWH or falls prevention practices for this population, especially in sub-Saharan Africa where the greatest burden of HIV exists [14]. Studies regarding physiotherapists’ knowledge, attitudes and practice patterns in falls prevention were among older adults of the general population [15–17], and stroke patients [18]. Physiotherapists’ perceptions on provision of fall prevention and bone health services were explored in the context of primary care of the general population [19–21]. Physiotherapists play a crucial role in falls prevention in at-risk populations [22]. An evaluation of their current knowledge and practices is an important step in ensuring conformity to best practice [23]. This study therefore aims to explore physiotherapists’ awareness of accelerated bone demineralisation and fall risk, and current prevention practices for PLWH in four sub-Saharan African countries. Recommendations from this study may inform physiotherapists and other health care providers involved in the primary care of PLWH, resulting in physiotherapy being valued as an integral component of primary HIV care. It may also form the basis for knowledge translation research for physiotherapist community to conform to evidence-based practice in their care of PLWH, thus improving their health service delivery to this vulnerable population.

## Methods

### Design

An exploratory descriptive qualitative study was used since little is known about this topic. Ethical approval was obtained from the Health Research Ethics Committee of Stellenbosch University (S18/07/137) and permission to conduct the study granted from the health professions or research regulatory bodies in all four countries. Written informed consent was obtained from all participants prior to the interviews. This paper reports on a subset of interview data collected regarding physiotherapists’ awareness of fall risk and accelerated bone loss in PLWH. The other interview data has been used to report on health care system challenges affecting falls prevention in PLWH and has been reported in another paper [24]. The report followed the consolidated criteria for reporting qualitative research (COREQ) [25].

### Research team

All investigators were physiotherapists and had experience in qualitative research.

### Study participants and recruitment

We purposively sampled physiotherapists who had at least two years’ experience in the primary care of PLWH and were currently working in public primary care facilities in the urban districts of the capital cities of four sub-Saharan African countries (Botswana, South Africa, Zimbabwe, and Zambia). Physiotherapists were excluded if they were not currently registered with their respective physiotherapy boards/councils and if they did not provide written consent. Randomised lists of primary health care (PHC) facilities from each selected district were created using the automated function in Microsoft Excel. Following these lists, the randomly selected PHC facilities were telephoned to identify physiotherapists who met the inclusion criteria. Participant information booklets and consent forms, in which the nature and purpose of the study was fully explained, were electronically mailed to willing participants. Participants who returned their signed consent forms were further contacted to arrange for an interview within a month’s timeframe.

According to recommended sample size for phenomenological studies [26], it was proposed a priori that at least 5–6 participants per country would be interviewed. However, purposive sampling prescribes continued sampling until data saturation is achieved, that is until no new significant information is obtained [27].

### Data collection and analysis

Telephonic in-depth interviews took place between December 2018 and July 2019 at pre-appointed times that would not interrupt the participants’ clinical practice. All interviews were conducted by the primary investigator (PI) although a second interviewer (KB or QL) was present to ensure consistency and coherence. An interview guide (see Additional file 1) was designed according to similar qualitative studies of rehabilitation specialists’ perceptions of falls prevention for the general geriatric population in primary care settings [21, 28] (Table 1).

**Table 1:** Sample of interview questions

Tell me about your experience of treating PLWH. What key functional problems do they present with?	
What is your understanding of the risk of falling among PLWH?	
What is your understanding of the effect of ART on bones in PLWH?	
Have any of your patients living with HIV presented with complaint of falls? What are the common reasons?	
How do you assess for falls in your routine care of PLWH?	
What are you currently doing for falls prevention in your delivery of care to PLWH?	

Further probing questions were used to clarify and gain deeper understanding of responses. No pilot interviews were conducted. However, new relevant issues that arose during preceding interviews were addressed in subsequent interviews. For example, participants reported inadequate undergraduate training resulting in them not being skilled in falls prevention. Therefore, subsequent interviewees were asked questions about their undergraduate training, postgraduate training, and continuous development opportunities regarding falls prevention in PLWH.

Interviews were recorded electronically and transcribed verbatim with the assistance of professional transcribers. The PI transcribed five transcripts to allow immersion into the data and develop the skill [29]. Transcripts were returned to participants to check that transcribed accounts accurately reflected what they had said.

Thematic content analysis with an inductive reasoning approach was applied [30]. Three transcripts were independently coded by three members of the research team (MC, KB, QL) by repeatedly reading transcripts to identify common conceptual themes and patterns. Data triangulation was done by consulting notes taken by interviewers as well as the reflexive notes recorded by the PI. Differences were discussed until consensus was reached. A codebook was created, which was applied to the rest of the transcripts with the aid of qualitative data analysis software Atlas.ti.8®. Analysis was an iterative process involving repeated cycles of data collection, transcription and analysis [27].

## Results

A total of 30 physiotherapists were invited to participate in this study. Seven physiotherapists declined to participate citing inadequate knowledge regarding the topic while one declined due to lack of time. Two physiotherapists who had given consent to participate could not be contacted on the scheduled appointments despite follow-up. Twenty-one interviews were conducted over a period of seven months. The length of interviews ranged from 22 to 30 min. Table 2 outlines the participants’ sociodemographic descriptions.

**Table 2:** Participants’ characteristics

Variable	Number (***n*** = 21)	Percentage (%)
**Gender**		
Male	4	19
Female	17	81
**Work setting**		
General or Referral Hospital	13	62
District Hospital	6	29
Military Hospital	1	5
Primary Health Clinic	1	5
**Cities (Countries)**		
Gaborone (Botswana)	6	29
Cape Town Metropole (South Africa)	4	19
Lusaka (Zambia)	6	29
Harare (Zimbabwe)	5	24
**Professional Qualification**		
BSc. Physiotherapy	17	81
MSc. Physiotherapy	4	19
**Age in years (mean ± SD; IQR)**	33.76 ± 4.22; 26–43	
**Years of professional experience (mean ± SD; IQR)**	9.67 ± 4.22; 3–19	
**Years of caring for PLWH (mean ± SD; IQR)**	8.90 ± 4.34; 3–19	

### Main findings

Three themes describing physiotherapists’ poor awareness of falls risk and bone demineralisation in PLWH and suboptimal fall prevention services were identified (Table 3). Themes, categories, and verbatim supporting quotations are presented below.

**Table 3:** Themes and categories identified from interview data

Theme	Category
Physiotherapists (un) awareness of falls risk	Not anticipatory of falls in PLWH
	Falls associated with geriatric population
	Unaware of serostatus of PLWH
	Minority of physiotherapists aware of falls in PLWH
Physiotherapists (un) awareness of bone demineralisation in PLWH	Unaware of effects of HIV or ART on bones
	Inaccessible facilities for BMD measurement
	Minority of physiotherapists aware of bone demineralisation in PLWH
Suboptimal fall prevention services	Fall risk assessment not prioritised
	Inadequate primary fall prevention strategies
	No screening or assessment tools available
	Inadequate referral to multidisciplinary team by physiotherapists

### Theme one: physiotherapists’ (un) awareness of fall risk in PLWH

Most physiotherapists expressed that they were not aware of the potentially inherent fall risk that could be present in PLWH. The physiotherapists acknowledged that they had not been anticipative of falls in PLWH and had never thought of assessing falls in this population.

*‘It’s difficult for me to explain that question directly … but specifically to say that this person is HIV positive hence they have this risk of falls, I haven't really observed that.’* Participant 14, Zambia.

Some still expressed some level of uncertainty towards this phenomenon being evident in PLWH. Conversely, some who had been initially unaware of falls in PLWH were able to, in retrospect, conclude that falls were a problem in PLWH.

Most participants perceived falls as a geriatric condition that is not particularly characteristic in PLWH.

*‘ … because mostly when we are talking about falls, we are talking about it in the elderly … but never have you ever heard an emphasis being put on falls with people living with HIV.’* Participant 21, Zimbabwe.

A few physiotherapists shared that patients were sometimes unaware of their HIV status because of not routinely testing for HIV. Sometimes patients who were aware of their seropositive status did not reveal it to their physiotherapists, especially when the therapist did not inquire about it. Therefore, the physiotherapists could not always associate presenting comorbidities and impairments with HIV or ART during assessment.

However, less than a third of the physiotherapists, mostly from Botswana and South Africa, recognised the problem of falls in PLWH. Falls were observed in hospitalised PLWH more than community-dwelling, with variable fall rates estimated between 5 and 60%. A few physiotherapists were aware of the risk factors for falls in PLWH, mostly attributing it to balance impairments (due to impaired sensation and loss of proprioception), frailty, muscle wasting, dizziness, ART non-adherence and comorbidities such as depression, tuberculosis, cerebral meningitis, stroke, hypertension, Kaposi Sarcoma and peripheral neuropathy. Two physiotherapists were aware of the negative impact of falls on the lives of PLWH, mentioning consequences such as fear of falling and fall-related fragility fractures.

### Theme two: physiotherapists’ (un) awareness of bone demineralisation in PLWH

Most physiotherapists were not aware of the effect of HIV infection or ARVs on increased risk of bone demineralisation in PLWH. They were aware of other side effects of ART such as lethargy, paraesthesia and myalgia but not accelerated bone loss.

*‘Some of my patients have presented with just general complaints after starting ARV’s maybe, they are feeling tired, they are feeling weak and also like general joint pain or body pains as well … I can’t say that it has been on their bones no.’* Participant 8, South Africa.

***‘****I don’t have enough understanding on that aspect. I only know that ART can have an effect on the nerves … On the nerves, yes, I know, but on the bones, I am not so much sure.’* Participant 11, Zambia.

Some felt that it would require a bit of research to compare the differences in BMD of PLWH and seronegative patients. All the facilities did not have dual energy x-ray absorptiometry (DXA), considered the gold standard for BMD measurement. Most used normal x-rays as their main diagnostic tool for osteoporosis with limited use of computer topography due to its high cost.

A minority of the participants (*n* = 3) supported the fact that ART or HIV infection itself can have a negative impact on BMD, having observed osteoporotic bones on X-rays of PLWH. A few cases of pathological fractures in PLWH were also reported. Some participants attributed bone loss in PLWH to effects of prolonged bed rest rather than effects of ART or HIV infection itself.

### Theme three: suboptimal falls prevention services

All the physiotherapists perceived their current fall prevention practice as suboptimal. This theme described factors mentioned by the physiotherapists that supported this notion.

#### Fall risk assessment not prioritised in PLWH

Because they were not aware that PLWH had a high risk of falls, most participants did not routinely assess for falls risk in this population. They prioritised other conditions that PLWH presented with for physiotherapy management. Very few physiotherapists subjectively asked about falls history in PLWH. The physiotherapists were not carrying out multifactorial risk assessments, especially in patients who had not presented with complaints of falls, even though they were able to identify patients with balance impairments during traditional assessments.

*‘I haven’t been doing that much, you know assessing the risk of fall, really unless if it's in the elderly patients, perhaps at the end of neuro rehabilitation just as a screen to see if this patient needs more rehabilitation or not, but in general we don’t really assess so much of the risk factors.’* Participant 2, Botswana.

One physiotherapist expressed concern regarding the high workload that would result if she did her own routine assessment and preferred to only assess fall risk in patients who had been referred by the doctors. It was also reported that doctors rarely referred PLWH specifically for falls prevention.

#### Inadequate primary falls prevention strategies

Most participants reported that they did not have any primary prevention strategies but focused more on secondary prevention for patients who have already experienced falls.

*‘I don’t think we have anything in place for falls prevention. I think we only start treatment when we find that someone has been falling a lot.’* Participant 4, Botswana.

**Fig. 1:**
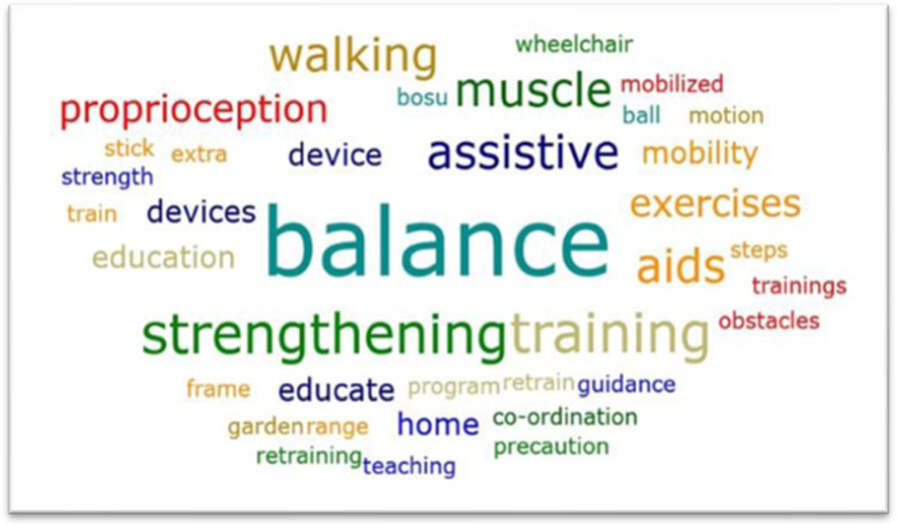
World cloud showing that the most mentioned fall prevention strategies currently employed by the participants were secondary fall prevention strategies

Common secondary prevention strategies employed in their current management of falls among PLWH included balance retraining, muscle strengthening, recommending, and training use of assistive devices (Fig. 1).

#### No screening/assessment tools available

Lack of rehabilitation-specific generic falls assessment tools in their facilities to guide them in their provision of care to any patient, let alone tools specific to PLWH, emerged as one of the hindrances to fall prevention practice. It was left to individuals’ discretion to decide which tool to use for assessment of falls in PLWH.

*‘Usually, it’s not us who do the screening. It’s usually done in the wards by the nurses, but we don’t have anything in place to use to assess risk of falls, but there should be. There are a couple of tools that could be put in place but currently we are not using anything … Actually, there is nothing in my department so it’s up to an individual to look for what works best for them.’* Participant 5, Botswana.

The Berg Balance Scale (BBS) was the most mentioned outcome measure used for assessment of balance, which the physiotherapists carried out as part of their generic assessment without specifically assessing for falls.

#### (Inadequate) referral to multidisciplinary team by physiotherapists

Some physiotherapists were aware of the need to involve other health care professionals in their management of falls risk. Health care professionals mentioned included dieticians, ophthalmologists, psychologists, social workers, and occupational therapists. However, two participants did not see the relevance of referring to other health care professionals once the patient had been referred to them.

*‘From what I recall we would be the end point … ’* Participant 17, Zimbabwe.

*‘Inasmuch as I know people refer these patients to me so that I prevent the falls. I get referrals and then I address the issue. I don’t refer to anyone else cause they would have been passed onto me.’* Participant 20, Zimbabwe.

## Discussion

This is the first study to explore the awareness by physiotherapists working in primary care facilities in sub-Saharan Africa of falls risk and bone demineralisation in PLWH and current fall prevention practice. The physiotherapists lacked adequate awareness of falls risk and bone demineralisation among PLWH. They did not necessarily link falls or fragility fractures to HIV or ART and deemed fall prevention services to be sub-optimal. The findings from this study have several implications for physiotherapy practice.

### Improve awareness of falls among PLWH by physiotherapists

The study results demonstrate a need for physiotherapists to be more anticipatory of the risk of falls and bone demineralisation when assessing PLWH. Most physiotherapists were not aware and therefore did not consider falls when assessing PLWH. The main reasons PLWH were referred to physiotherapy were linked to comorbidities such as tuberculosis, cerebral toxoplasmosis, Kaposi sarcoma and polyneuropathies that resulted in pain, altered function, and reduced QoL. The physiotherapists would often prioritise the pain, motor deficits and respiratory dysfunctions. The risk of falls and related fractures were often assessed as a safety precaution before mobilising patients with obvious balance and co-ordination problems. No comparable studies were identified on awareness of falls in PLWH and physiotherapists (85%) seem to be more aware of falls in other at-risk populations, such as stroke survivors [18] and geriatrics [17]. Three cross-sectional studies [31–33] in high-income countries reported falls prevalence ranging from 18.6–40.7% in middle-aged to older PLWH. In LMIC, falls may be prevalent in younger cohorts of PLWH due to the different socio-demographic profile of PLWH or more virulent strains of Clade C HIV [34]; one recent South African study [35] attesting to this possibility. Hence, while Greene et al. [4] recommended routine screening for falls in all PLWH who are 50 years and older, physiotherapists may need to assess for falls in both younger and older PLWH in LMIC.

Most participants’ responses were inclined towards hospitalised PLWH post-fall, while information about community-dwelling PLWH mostly emerged after probing. By considering physiotherapists working in primary care settings for inclusion in this study, it was expected that most responses would concern primary falls prevention among community-dwelling PLWH. This finding could mean that less falls occur in community-dwelling PLWH compared to hospitalised PLWH. On the other hand, community-dwelling persons may have experienced more falls than hospitalised persons (perhaps due to greater exposure to external factors contributing to falls e.g. outdoor falls resulting from uneven terrain and vigorous activity) [36]. However, the physiotherapists may possibly be less aware of falls occurring in community-dwelling PLWH because people who fall but do not require healthcare may not report the falls without being asked specifically [37]. While post-fall assessment is important in identifying the cause of prior falls and prevention of further falls [38], risk-screening for falls and education of communities regarding prevention may prevent complications of falls such as injuries, fractures, fear of falling and disability [39].

The need for physiotherapists to be wary of falls in PLWH is further augmented by the finding that physicians rarely referred patients specifically for falls management, but rather for mobility. Chou et al. [40] reported referral of patients with unsteady gaits to physiotherapy as a facilitator to falls risk management. A recent systematic review [41] concluded that PLWH may have gait impairments reflective of fall-related parameters in older persons. This places the responsibility on physiotherapists to routinely screen for falls in all PLWH referred for mobility management as well as refer appropriately to other health care professionals.

### Increase use of standardised screening tools and outcome measures

Although some participants were aware of the risk factors for falls in PLWH, most did not use standardised screening tools or outcome measures to identify and assess PLWH at risk of falls. A few participants used the BBS as an outcome measure in balance assessments. Physiotherapists’ inconsistent use of screening tools for falls has been documented. For example, although 56.9% of the physiotherapists in one Belgian survey [18] acknowledged the need for fall-risk evaluation at the beginning of treatment, only 32.3% used standardised outcome measures in screening for falls among their stroke patients. The use of outcome measures is important in evaluating treatment progression as well as demonstrating the effectiveness of physiotherapy interventions to relevant stakeholders and policymakers. More research is needed to determine the most predictive tools for assessing risk factors for falls in PLWH.

With regards to screening for BMD loss, most participants were not able to access Dual-energy X-ray absorptiometry (DXA), a tool most predictive in identifying patients at high fall risk because of BMD loss [42]. The physiotherapists mostly relied on less precise X-rays; perhaps because they were more available and less expensive. One study [43] demonstrated calcaneal quantitative ultrasound (QUS) as a feasible alternative to DXA in screening BMD in PLWH, and Berner et al. [35] used it successfully in a resource-limited setting. Perhaps primary health facilities can consider investing in this ‘cost effective, portable and ionizing-radiation free tool’ [2] to enable PLWH to be routinely screened for bone loss.

### Differences between the countries

A few differences were noted between the countries and health care settings that may have influenced the perspectives of the physiotherapists about the risk of bone demineralisation and falls in PLWH. Botswana and South Africa, both upper-middle income countries, had a few physiotherapists (less than a third) who were aware of falls in hospitalised PLWH. These were mostly from facilities that had gone through the hospital accreditation process which may have reinforced fall prevention as part of patient safety [44]. Another difference was that Botswana outsourced physiotherapy training from high-income countries such as Australia and Ireland [45], where HIV is not priority. This again may have contributed to the physiotherapists’ lack of awareness and sub-optimal fall prevention practice for PLWH. However, despite these factors, it remained evident that physiotherapists were generally unaware of the risk of falls and bone demineralisation in PLWH, more so in community-dwelling PLWH.

### Limitations of the study

By considering physiotherapists working in urban districts of the selected cities, the results of this study may not be generalised to physiotherapists working in rural primary HIV care settings where different health system structures and patients with different socio-demographic profiles may exist. Also, our sample may have been biased towards a more knowledgeable sample since some participants declined participation in the study because they felt they had insufficient knowledge about the subject.

Further research is needed to establish the magnitude of certain problems highlighted in our study by quantification of data. For example, a quantitative survey would determine what proportion of physiotherapists lack awareness of falls and bone demineralisation in PLWH and therefore whether there is a need to employ interventions to raise an awareness among physiotherapists.

Word clouds (which are based on the concept of font size being proportional to the frequency of word usage) may not have been the most accurate way to display the current falls prevention strategies as they conflate the number of participants who used a word and the number of times all participants used a word [46]. However, word clouds offer a quick means of visual exploratory data analysis that is easy to interpret and display [47]. Thus more important than a count of the proportion of physiotherapists who mentioned a strategy, the word cloud served the purpose of highlighting at a quick glance that the most mentioned strategies were secondary prevention practices.

## Conclusion

Findings from this study indicate that physiotherapists in sub-Saharan Africa are not aware of the potential risk of falls and bone demineralisation in PLWH, and thus do not routinely assess for these phenomena in both older and younger PLWH. Meanwhile, more research is evidently needed regarding falls in PLWH and the effects of BMD loss on falls in PLWH, especially in sub-Saharan Africa. This evidence may form the basis for revision of undergraduate rehabilitation curricula and continuous professional education needed to improve awareness and delivery of care among physiotherapists and other health professionals involved in primary HIV care. Evidence-based practice will ultimately result in improved health care outcomes and quality of care for PLWH.

## Supplementary Information

**Additional file 1.**

## Data Availability

The participants involved in this study can be identified through the interview transcripts, even after names are removed, because of the few physiotherapists working in primary care. The datasets used and/or analysed during the current study are available from the corresponding author upon reasonable request.

## Abbreviations

ART: Anti-retroviral therapy
BBS: Berg’s balance scale
BMD: Bone mineral density
DXA: Dual-energy X-ray absorptiometry
HIV: Human immune-deficiency virus
LMIC: Low-to-medium income countries
PLWH: People living with HIV
QoL: Quality of life
QUS: Quantitative ultrasound
