# Supplementary material for: Physiotherapists’ awareness of risk of bone demineralisation and falls in people living with HIV: a qualitative study
Source: BMC Health Serv Res. 2021 Apr 13;21:333. doi: 10.1186/s12913-021-06343-1 (PMC8045224; doi:10.1186/s12913-021-06343-1)
Supplement: Supplementary file 1 — Additional file 1. [file 12913_2021_6343_MOESM1_ESM.docx]

## Interview schedule

**Demographic details (obtained via email or WhatsApp):**

1. Age

2. Educational Background

3. Work experience

4. Workplace

**Interview questions:**

**Please help me better understand your experience of addressing issues related to falls and fall related fractures in PLWH.**

**Icebreaker:** Tell us about your experience of treating PLWH. (How long? Proportion of total patient load? Which age group do most of them belong?)

**Introductory question:** What key functional problems do PLWH present with to rehabilitation? What do patients say are their functional limitations?

**Key questions:**

Have any of your patients living with HIV presented with complaint of falls? Pathological fractures? What are the common reasons?

What is the difference between the prevalence of falls in PLWH who are hospitalised and those who are living in the community?

What is your understanding of the risk of falling among PLWH?

What are the differences in presentation of patients on and not on ART?

What is your understanding of the effect of ART on bones in PLWH? On movement?

How do you assess for falls in your routine care of PLWH? Which screening tool do you use to identify PLWH who you suspect are at high risk of falls? What determines your choice of tool?

What are you currently doing for falls prevention in your delivery of care to PLWH?

Which Clinical Practice Guidelines for falls prevention do you refer to?

Which health care professionals do you refer your patients to for further falls risk prevention?

What post-graduate training on falls prevention/ care of PLWH have you received? What training workshops/opportunities are available on falls prevention in PLWH? Was emphasis put on functional limitations or was the focus on pathology of HIV in your undergraduate training?

Any recommendations regarding what physiotherapists need to do improve care of PLWH considering their potentially high risk of falls and reduced BMD?

**Ending question:** Is there anything else you which I have not asked you which you would like to share with me?

**My reflections on the interview process:**
